# Moving beyond moderate-to-vigorous physical activity: the role of light physical activity during adolescence

**DOI:** 10.3389/fspor.2023.1282482

**Published:** 2023-11-02

**Authors:** Deborah M. Telford, Rebecca M. Meiring, Silmara Gusso

**Affiliations:** Department of Exercise Sciences, University of Auckland, Auckland, New Zealand

**Keywords:** physical activity, sedentary behaviour, light intensity, time-use, movement behaviours, adolescence, youth

## Abstract

Regular physical activity is an important component of a healthy lifestyle for young people. However, an estimated 80% of adolescents globally are insufficiently active. Traditionally, health benefits were attributed only to physical activity of at least moderate intensity, and recommendations focused on achieving a threshold of moderate-to-vigorous physical activity, without consideration of other aspects of movement within the 24 h cycle. Recently, the overall daily balance of active and sedentary behaviours has gained recognition as an important determinant of health. However, the relationship between light intensity physical activity and health has not been fully explored. In this perspective paper, we discuss key challenges in defining, measuring and analysing light physical activity which have hindered the advancement of knowledge in this area. Next, we suggest three ways in which light physical activity may enhance adolescent wellbeing: firstly, by replacing sedentary behaviours to increase daily movement; secondly, by supporting the accumulation of higher intensities of physical activity; and thirdly, by providing positive experiences to facilitate lifelong engagement with physical activity. In highlighting the importance of light physical activity during adolescence, we aim to encourage critical reflection and the exploration of new approaches towards physical activity within public health and beyond.

## 1. Introduction

Regular physical activity during adolescence is beneficial for many aspects of health, including cardiorespiratory fitness (1), bone health (2), cardiometabolic health (3), body composition (4) and mental health (5). However, the time spent in physical activity declines throughout adolescence at an average rate of approximately 6%–7% per year (6, 7) and an estimated 4 out of 5 adolescents worldwide are insufficiently active (8). As those who are physically active during adolescence are more likely to engage with physical activity later in life (9, 10), establishing an active lifestyle during this life-stage can positively impact long-term health trajectories into adulthood, where physical inactivity is a leading cause of morbidity and mortality (11). Therefore, it is essential to prioritise adolescent physical activity on the global health agenda.

Efforts to combat physical inactivity in youth have primarily centred around the accumulation of 60 min of daily moderate-to-vigorous physical activity (MVPA), in accordance with guidelines (12). However, interventions targeting MVPA in adolescents have been largely ineffective (13, 14). Furthermore, growing evidence on the adverse effects of excessive sedentary behaviour throughout the day has challenged the traditional MVPA-focused perspective, suggesting that other aspects of movement, such as light physical activity (LPA), might also play a crucial role in shaping overall health and wellbeing throughout the lifespan (15, 16).

Engaging in LPA may be an attractive alternative to MVPA for adolescents who are reluctant or unable to take part in higher intensity physical activity. Fatigue and sleepiness are prevalent issues during adolescence, affecting 30%–40% of healthy teens (17), and lack of energy is a commonly reported barrier to physical activity (18). LPA has a lower energy cost relative to MVPA, and may present less of a barrier amongst those with low energy. Additionally, LPA can be readily accumulated through incidental activities, providing a way to increase daily movement outside of structured physical activity programs. Whilst sports participation can be of immense value during adolescence (19), it does not appeal to all, and may be challenging for some. For instance, engagement is typically low amongst adolescents who experience mental health problems (20). Increasing time spent in LPA may provide a more accessible way to reap the benefits associated with physical activity for adolescents who face participation barriers.

In recent years, research has shifted towards exploring the health impact of movement behaviours throughout the whole day (24 h behaviours), encompassing all physical activity, sedentary behaviour and sleep (21, 22). Several countries have adopted 24 h activity guidelines for youth which recommend a healthy balance of 24 h behaviours, including several hours a day of LPA (23–25). In adults, clear benefits of LPA for health are beginning to emerge. In a meta-analysis of data from 15 cohort studies, LPA was linked to a reduction in all-cause mortality, with a 40% reduced risk for those with high LPA compared to those with low LPA (26). However, the health impact of increasing time spent in LPA relative to other 24 h behaviours during adolescence is less well understood (27). This knowledge is essential to our understanding of a healthy 24 h day.

In this paper, we highlight the potential importance of LPA for adolescent health and wellbeing. We discuss current challenges associated with the measurement and analysis of LPA, especially the limitations of common measurement tools in distinguishing LPA from sedentary behaviour or standing. In light of these issues, we contend that the health benefits of LPA in adolescents remain insufficiently explored. Additionally, we present a perspective on LPA as an essential component of human movement, integral to the accumulation of MVPA and the development of a lifelong positive relationship with physical activity.

## 2. Definition, measurement and analysis of LPA

LPA is defined by the World Health Organisation as a subset of physical activity which is performed between 1.5 and 3 metabolic equivalents (METs) (12), where one MET is the rate of oxygen consumption while sitting at rest (28). By this definition, LPA encompasses bodily movements produced by skeletal muscles requiring energy expenditure, but which do not substantially increase the heart rate or breathing rate (12, 28). Examples of LPA include doing household chores, playing active video games and slow walking (29).

Another definition of LPA has emerged from 24 h time-use paradigms, which propose a distinction between sedentary behaviour (waking behaviours in a sitting or lying posture with energy expenditure ≤1.5 METs) and physical activity (all other waking behaviours) (30). Accordingly, LPA is expanded to include not only movement performed at 1.5–3.0 METs but also activities performed standing still (21) which might include standing while watching TV or playing non-active video games (29). Whilst this definition supports a parsimonious framework of 24 h activity, it presents a challenge in LPA research, since incorporating standing and moving within a single category may obscure differences in their effects (31). An alternative framework, proposed by Pedišić (32), treats standing as a separate category within the 24 h cycle, distinct from LPA and sedentary behaviour. However, this approach has not been widely adopted, and requires measurement tools capable of assessing both posture and movement.

Accurate assessment of LPA is crucial but challenging. Questionnaires lack the precision to capture incidental or lifestyle physical activity, making them unsuitable for LPA measurement (33). In contrast, accelerometers can assess movement throughout the day. However, commonly used devices positioned at the hip, waist or wrist lack the capacity to distinguish between different postures, such as standing and sitting (34). Instead, 24 h behaviours are estimated using accelerometry cut-points. Whilst this method provides a reliable assessment of MVPA, estimation of LPA is less precise (35), and standing time cannot be clearly distinguished from sedentary behaviour or LPA.

An alternative class of accelerometers, worn on the thigh, offer distinct advantages for LPA measurement. Devices designed for this wear location can distinguish between different postures and activities such as lying, sitting, standing and stepping, whilst also assessing movement (36). Despite these benefits, thigh-worn accelerometers are underutilised in adolescent research, perhaps due to a lack of validated cut-points for the estimation of MVPA with these devices (37). Greater adoption of methods which allow standing time to be measured separately from LPA would be advantageous to the study of LPA and its relationships with health.

Analysing relationships between LPA and health outcomes presents further challenges, due to the complex nature of 24 h behaviours. Multicollinearity is a fundamental issue, as changes in one behaviour must be offset by changes in others. For example, if sleep duration is reduced, there must be a compensating increase in waking behaviours. Traditional statistical methods, such as regression models, assume independence between variables, making it difficult to establish the health benefits of one behaviour whilst controlling for others. Recently, compositional data analysis (CoDA) has been proposed to address this issue (38, 39). With this method, 24 h behaviours are expressed in relative terms as proportions of a whole day, and log-ratios are calculated which reflect the time spent in one behaviour relative to others. These log-ratios can then be analysed using traditional statistical methods without violating independence assumptions.

LPA’s unique position along the intensity spectrum between sedentary behaviour and MVPA adds further complexity. As changes in LPA can be offset by changes in either sedentary behaviour or MVPA, greater LPA can reflect either a more active or less active mix of 24 h behaviours. As a result, the health impact of higher levels of LPA can be difficult to interpret if the behaviour being replaced by LPA is not defined. This presents a problem when using traditional multivariate regression. Isotemporal substitution is a method of analysis which offers a potential solution to this problem (40). With this method, all behavioural components are included in a regression model bar one. The regression coefficients represent the effects of replacing each behaviour in the model with the behaviour that was excluded from the model. Recently, compositional isotemporal substitution methods have been developed, providing ways to estimate the health impacts of reallocating time between behaviours within a CoDA framework (41). The development and use of new methods such as CoDA and isotemporal substitution within time-use epidemiology offers great promise for furthering our understanding of relationships between LPA and health, provided that LPA can be measured accurately.

## 3. LPA and health

A large body of evidence supports the health benefits of MVPA during adolescence (42). However, relatively little is known about the health impact of LPA, which has been much less widely studied (43). The effect of replacing sedentary behaviour with LPA is of particular interest. Currently, evidence on the physiological effects of sedentary behaviour in adolescents is relatively limited. However, in adults, there is extensive evidence linking sedentary behaviour to chronic diseases and early mortality, and studies indicate that excessive sedentary behaviour can have adverse physiological effects even when MVPA is above guidelines (44). As chronic health problems are rising in younger populations (45), it is vital to understand how the balance of LPA and sedentary behaviour during adolescence might influence health outcomes.

The available evidence regarding LPA and health in adolescents is weakened by methodological challenges. Failure to adequately control for other 24 h behaviours has been a pervasive issue in time-use studies (32) and standing time is very rarely measured or reported separately from LPA. Additionally, use of a variety of accelerometry cut-points has resulted in inconsistent measures of LPA (37) and studies have often failed to address the complexities arising from LPA’s position on the intensity spectrum, examining the effects of LPA *per se* rather than the effects of replacing sedentary behaviour with LPA (43). Nevertheless, existing evidence may offer clues to LPA’s relationship with health during adolescence.

Two health outcomes which have been relatively widely studied in relation to physical activity of differing intensities are cardiometabolic health and obesity. In a 2016 review of physical activity and health in school-aged youth, favourable associations were found between LPA and some cardiometabolic health markers, including diastolic blood pressure, blood pressure z-score, insulin resistance and HDL cholesterol (43). Recent studies using isotemporal substitution also support the hypothesis that LPA may be beneficial for cardiometabolic health in adolescents (46, 47).

Concerning obesity, evidence for associations with LPA is highly inconsistent, with a mixture of favourable, adverse and null findings in studies prior to 2016 (43). Similar conflicting results are seen in recent studies using CoDA (48, 49) and isotemporal substitution (50–52), with adverse (48, 51), null (52), mixed (49), and favourable (50) results.

Methodological issues in LPA measurement may contribute to adverse and inconsistent findings in obesity research, in addition to inconsistent measures of obesity and confounding factors such as diet (53). In adults, a study comparing relationships between adiposity markers and LPA assessed with thigh-worn and hip-worn accelerometers found opposing results between the two assessment methods, where associations were positive with thigh placement and negative with hip placement (54). To the best of our knowledge, only one study has examined LPA and obesity in adolescents using thigh-worn accelerometers (55). In this study, greater LPA was associated with lower BMI and skinfold thickness after adjusting for MVPA, and associations were stronger when standing time was excluded from LPA. These findings indicate that relationships between LPA and adiposity may be dependent on how LPA is conceptualized and assessed, highlighting the need for precise, clearly defined measurement methods.

Initial evidence suggests possible benefits of increasing LPA for certain health outcomes, particularly cardiometabolic health. However, very little is known about the impact of physical activity of different intensities on other important outcomes, including mental health (56, 57) and quality of life (58, 59) where few studies have focused on LPA. Despite advances in analytical methods, many studies continue to rely on more established methods such as traditional linear regression. Evidence is further limited by use of measurement tools and protocols optimised for assessing MVPA, which lack the capacity to measure LPA separately from standing time. Whilst isotemporal substitution is now often used to assess the effects of replacing LPA with other behaviours, to the best of our knowledge this method has not yet been applied using data collected with thigh-worn accelerometers in adolescents, which potentially provide a more precise, well-defined measure of LPA. Thus, the current state of evidence on the relationships between LPA and health during adolescence remains limited, with a lack of robust data.

As a growing body of research is focused on examining the effects of substituting one behaviour with another, attention should now turn to contexts within which these substitutions might occur, and how 24 h behaviours interact in real life situations, so that these findings might be used to inform realistic behaviour change strategies. Currently, there is evidence that shorter sleep duration in children and adolescents is primarily offset by increased sedentary behaviour, rather than physical activity (60). However, interactions between waking behaviours such as the impact of changes in LPA on other 24 h behaviours remain unexplored.

## 4. LPA can support MVPA

LPA not only offers potential direct health benefits but may also play a crucial role in supporting the accumulation of MVPA. Whilst LPA and MVPA are commonly conceptualised as separate behaviour categories, this does not reflect the reality of free-living, where activities span a spectrum of intensities. For instance, in youth sports, MVPA accounts for 30%–60% of team games and practice sessions, and 10%–30% of individual sports sessions, suggesting a sizeable portion of training time is spent in LPA, standing, or at rest (61–64). Even activities involving continuous MVPA, such as long-distance running, typically begin and end with LPA to transition between rest and activity. Thus, LPA is intrinsic to structured physical activity and is necessary for the accumulation of MVPA within sports and leisure contexts. Additionally, time spent in LPA increases opportunities for unstructured, incidental bouts of MVPA. A slow walk to school can transition into a fast walk if the situation demands it, whereas this opportunity does not arise with motorised transport.

This potential coupling of LPA and MVPA may have implications for behaviour change strategies. For instance, efforts to substitute LPA for MVPA in order to reach the 60 min/day MVPA threshold might prove counter-productive. Instead, promoting lifestyle adjustments which increase LPA may be a fruitful approach, leading to increased opportunities for unstructured MVPA along the way. Walking interventions have shown promise in increasing step count in adolescents (65), providing initial support for this softer approach.

## 5. LPA can provide positive experiences

Efforts to determine the optimal volume and intensity of physical activity to maximise health benefits are important to inform public health policy and provide guidance for health professionals. However, promoting positive experiences with movement during adolescence, regardless of intensity, may increase future engagement with physical activity, leading to greater long-term public health benefits. Adolescents commonly report past negative experiences as barriers to participation in physical activity (61), indicating that the way people feel during and after physical activity is of vital importance.

Intensity is one factor which influences the way people feel during exercise, with a tendency for pleasurable feelings to diminish as intensity increases (66). In adults, studies indicate that feelings of pleasure tend to decline around the ventilatory threshold when the breathing rate increases at a rate disproportionate to oxygen uptake and steady-state activity can no longer be maintained (66). Comparable results have been found in adolescents, where most individuals reported displeasure during high-intensity exercise, and even at a moderate intensity of 80% of the ventilatory threshold, over 50% reported decreased feelings of pleasure (67). Additionally, when asked to select an exercise intensity that feels good, those with more favourable responses to moderate intensity exercise tend to select a higher intensity than those with neutral or negative responses (68).

These results indicate that affective responses to exercise differ amongst adolescents, and those who respond negatively at moderate intensity tend to spend less time engaging in MVPA (67). Thus, LPA may offer a unique opportunity to accumulate positive experiences for inactive adolescents who experience displeasure during moderate intensity exercise, although this is yet to be investigated.

## 6. Conclusion and future directions

In this brief perspective, we have highlighted ways in which LPA might contribute to a healthy 24 h day during the pivotal life-stage of adolescence. Whilst data on relationships between LPA and health during adolescence is scarce, this should not be interpreted as evidence of a lack of an effect, and further research is needed to illuminate the role of LPA for health and wellbeing in young people. Future studies should consider employing methods which incorporate postural assessment such as thigh-worn accelerometers, together with isotemporal substitution methods, to unravel the health impact of changing time spent in LPA relative to other behaviours, including standing.

We have argued that LPA’s contribution goes beyond direct health benefits, as LPA forms an integral part of human movement, and is necessary for the accumulation of episodes of higher intensity activity. Additionally, LPA during adolescence may help to generate positive experiences of physical activity, which may pave the way for greater future engagement.

To advance our understanding of a healthy 24 h day, we must now move beyond a focus on MVPA to examine the potential of all 24 h behaviours to benefit health and wellbeing. Here we have focused on the role of LPA. However, we should also consider how sleep and sedentary behaviours may contribute to wellbeing by, for example, helping adolescents to recover from mental and physical exertion and manage stress. Ultimately, by focusing on the short-term goal of increasing MVPA, we may risk missing critical opportunities for young people to progress towards a healthy balance of 24 h behaviours, now and throughout the lifespan.

## Data Availability

The original contributions presented in the study are included in the article. Further inquiries can be directed to the corresponding author.
